# The *Drosophila* ZO-1 protein Polychaetoid suppresses Deltex-regulated Notch activity to modulate germline stem cell niche formation

**DOI:** 10.1098/rsob.160322

**Published:** 2017-04-19

**Authors:** Hideyuki Shimizu, Marian B. Wilkin, Simon A. Woodcock, Alessandro Bonfini, Yvonne Hung, Sabine Mazaleyrat, Martin Baron

**Affiliations:** University of Manchester, School of Biological Sciences, Manchester Academic Health Science Centre, Michael Smith Building, Oxford Road, Manchester M13 9PL, UK

**Keywords:** Notch, endocytosis, *Drosophila*, signalling, ZO-1

## Abstract

The developmental signalling protein Notch can be proteolytically activated following ligand-interaction at the cell surface, or can be activated independently of its ligands, following Deltex (Dx)-induced Notch endocytosis and trafficking to the lysosomal membrane. The means by which different pools of Notch are directed towards these alternative outcomes remains poorly understood. We found that the *Drosophila* ZO-1 protein Polychaetoid (Pyd) suppresses specifically the Dx-induced form of Notch activation both *in vivo* and in cell culture assays. *In vivo* we confirmed the physiological relevance and direction of the Pyd/Dx interaction by showing that the expanded ovary stem cell niche phenotypes of *pyd* mutants require the presence of functional Dx and other components that are specific to the Dx-induced Notch activation mechanism. In S2 cells we found that Pyd can form a complex with Dx and Notch at the cell surface and reduce Dx-induced Notch endocytosis. Similar to other known activities of ZO-1 family proteins, the action of Pyd on Dx-induced endocytosis and signalling was found to be cell density dependent. Thus, together, our results suggest an alternative means by which external cues can tune Notch signalling through Pyd regulation of Dx-induced Notch trafficking.

## 1. Introduction

Notch is a transmembrane, cell–cell signalling receptor molecule associated with the apical epithelial junctions, and mediates an essential and highly conserved signal used in many developmental contexts to determine cell fate [1]. Notch activity is initiated through binding to a membrane bound ligand of the Delta/Serrate/Lag2 (DSL) family, which results in a cascade of proteolytic cleavages that eventually releases the Notch intracellular domain (NICD) [1]. The latter translocates to the nucleus to activate signalling through binding to the transcription factor Suppressor of Hairless. Notch can also be activated independently of its ligands through the activity of the ring finger ubiquitin ligase protein Deltex (Dx), which promotes Notch endocytosis and its activation within the endosomal pathway [2–4]. The latter depends on trafficking of Notch to the late endosome and is associated with the retention of Notch on the outer limiting membrane of this organelle. This maintains cytoplasmic accessibility of the NICD, which is released by a Presenilin-dependent proteolytic activation probably after endo-lysosomal fusion and removal of the Notch extracellular domain (NECD). The Dx-induced route to activation can be distinguished from ligand-induced signalling by the former's requirement for late endosomal trafficking components such as the homotypic fusion and vacuole protein sorting (HOPS) complex. For example, in *Drosophila,* mutations of the HOPS component *carnation* (*car*) prevent Dx-induced Notch activation but have no effect on ligand-induced signalling [2]. Dx also removes Notch from the cell surface, reducing its accessibility to ligands, and therefore Dx can act negatively on the ligand-induced form of signalling [4]. This context-dependency of Dx outcomes on Notch is exemplified by its interplay with the HECT domain ubiquitin ligase Suppressor of deltex (Su(dx)). The latter also binds to the NICD and promotes Notch transfer to a parallel clathrin-independent endocytic route and further promotes Notch entry into the internal luminal vesicles of late endosomes, resulting in Notch degradation [4]. Su(dx) and Dx are in competition with each other to direct Notch through these alternative routes. The phenotypic consequence of removing both Su(dx) and Dx in different cell types is context dependent. When combined, *dx* and *Su(dx)* mutations can be mutually suppressive for their respective loss or gain of Notch signalling phenotypes, as observed in the *Drosophila* wing. Alternatively the double mutant can produce enhanced Notch signalling through increased ligand-dependent activity, as observed in leg development and the embryo nervous system [4]. The latter situation can arise because both Su(dx) and Dx normally act to remove Notch from ligand accessibility by promoting Notch removal from the cell surface. The observed double mutant Notch gain of function can therefore occur through increased ligand-induced signalling.

Polychaetoid (Pyd) is the single *Drosophila* homologue of the cell junctional scaffolding protein ZO-1 [5] and has previously been implicated in the regulation of Notch signalling [6,7]. In mammalian epithelia ZO-1 is predominantly localized to tight junctions, where it forms complexes with junctional proteins such as Claudins that are responsible for establishing a barrier function. Hence cells deficient in ZO-1 and the close homologue ZO-2 fail to form proper tight junctions [8]. ZO-1 is also found in newly forming fibroblastic spot-like adherens junctions and may accelerate conversion to belt-like adherens junctions during epithelial polarization whereupon ZO-1 is relocalized to tight junctions [9,10]. ZO-1 is further present in adherens junctions of non-epithelial tissue [11]. In insects the tight junction is absent but functionally replaced by the more basally located septate junction. ZO-1 is predominantly localized to the adherens junction in *Drosophila* epithelia but is also found localized to lateral membranes [12]. Neither *Drosophila* nor mammalian ZO-1 are essential for overall epithelial cell polarity but both regulate apical domain expansion and morphology [7,13]. Furthermore in the *Drosophila* eye, *pyd* mutant cells accumulate the adherens junction proteins Cadherin and Roughest, and in wing disc epithelia Notch accumulation has additionally been observed, [7,14]. While ZO-1 is not required for cell polarity its loss has functional consequences on tissue morphology during development. In *Drosophila, pyd* mutants cause disruption during embryo morphogenesis through altered cell adhesion and cytoskeletal interactions resulting in defects in tracheal structures and dorsal closure [15,16]; *pyd* mutants further affect patterning of the eye through altered cell contacts [14]. In *Caenorhabditis elegans*, ZO-1 contributes to the strength of intercellular adhesion junctions, which are disrupted under stress of morphological movements during embryogenesis of *ZO-1* mutants [17].

ZO-1 also interfaces with components of the cellular signalling pathways to regulate proliferation and cell fate. For example mammalian (ZO-1) protein participates in a sensing mechanism that mediates cell density-dependent control of cell proliferation and cell signalling [18–20]. In *Drosophila, pyd* mutants affect wing growth through an interaction with the Hippo pathway and cell fate through misregulation of Notch signalling [7]. The *pyd* mutants result in either loss or gain of Notch activity depending on tissue context. In the notum of *pyd* mutants Notch activity is reduced, resulting in extra sensory macrochaetae. However in the ovary *pyd* mutants result in increased Notch activity, reflected in an enlarged stem cell niche [7]. *Su(dx)* mutants prevent the enlargement of the niche which occurs when the fly is heterozygous for a *pyd* null allele. This antagonistic genetic interaction may reflect a direct regulatory interaction since Su(dx) can form a complex with Pyd. Furthermore, the *Su(dx)* mutation does not suppress the homozygous null *pyd* phenotype suggesting that Su(dx) acts negatively on Pyd rather than vice versa [7]. How Pyd then impacts on Notch signalling in niche cells is not understood.

Here we found that *pyd* mutants upregulate the ectopic ligand-independent Notch signal induced by the overexpression of Dx in the *Drosophila* wing. We further found that functional Dx is required for full stem cell niche development and that *dx* mutants suppress the null *pyd* niche phenotype. The function of *dx* in the niche is contingent on the presence of *Su(dx)* since double *dx, Su(dx)* mutants produced the opposite outcome of an enlarged niche, accompanied by decreased Notch endocytosis. In contrast, the enlargement of the niche of *pyd* mutants was associated with efficient Notch endocytosis in developing ovaries. We suggest therefore that the niche expansions observed in these different genetic backgrounds are associated with different mechanisms of increased Notch activity, respectively through alternative ligand-dependent or Dx-dependent routes. Using *Drosophila* S2 cells in which different forms of Notch activity can be studied separately, we found that Pyd downregulated Dx-driven Notch signalling, but had little effect on ligand-activated Notch. We found that Pyd forms a complex with Dx and Notch at the cell surface and acts to suppress Dx-induced Notch endocytosis. As with other reported actions of ZO-1 proteins [18–20], we found that in cell culture Pyd was recruited to the cell surface at high cell density and acted in a density-dependent manner to suppress Dx-induced Notch endocytosis and signalling. Our findings therefore implicate Pyd as an upstream modulator of the late endosomal Dx-induced Notch activation mechanism and establish that, in principle, external cues can modulate Notch signalling levels via Dx regulation.

## 2. Results

### 2.1. Pyd suppresses Dx-induced Notch activation

**Figure 1.**
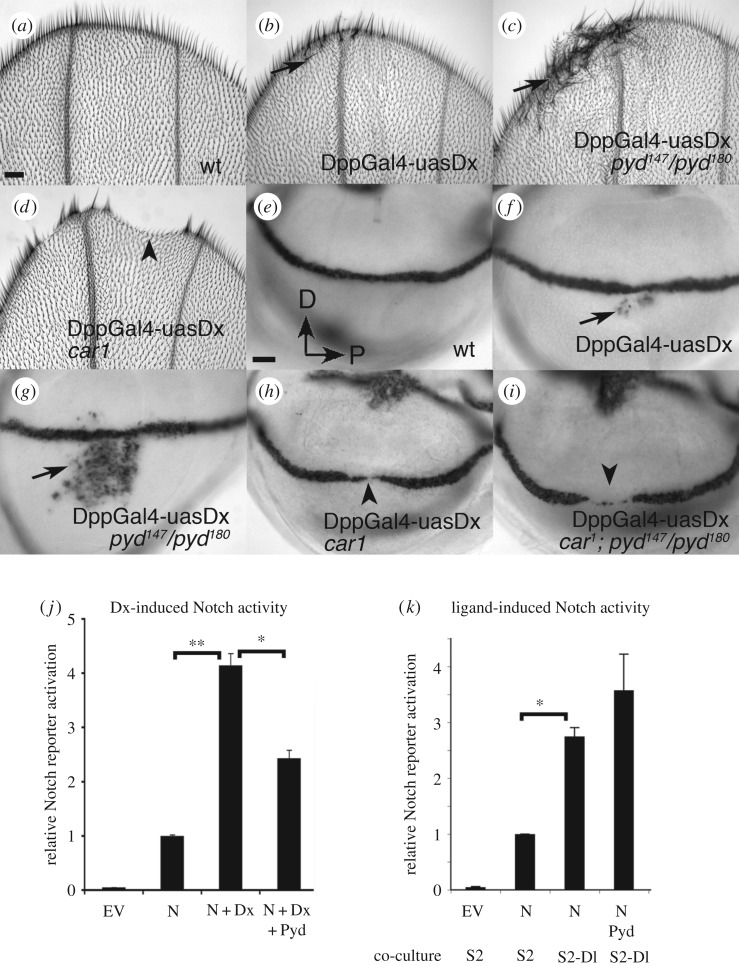
Pyd suppresses Notch signalling induced by Dx expression. Adult distal wing tips showing a wild-type (wt) margin (*a*), and ectopic margin bristles (arrows) induced by overexpression of Dx with Dpp-Gal4, in a wt background (*b*), or *pyd^147^/pyd ^180^* null (*c*). (*d*) shows notching associated with overexpression of Dx in a *car^1^* mutant (arrowhead). (*e–i*) Late third instar wing imaginal discs stained for *wingless mRNA* expression to report Notch activation. In a wt disc (*e*) there is a clear line of *wingless* expression demarcating the dorsal/ventral boundary. (*f–i*) Dx overexpressed along the A/P axis using the Dpp-Gal4 driver. (*f*) Mild ectopic *wingless* expression (arrow) induced in wt background. (*g*) Enhanced *wingless* expression in *pyd* null mutant. Overexpression of Dx in a *car^1^* mutant (*h*) or in a *car^1^*, *pyd* double mutant (*i*), leads to a loss of *wingless* (arrowheads). Scale bar in (*a*) represents 40 µm in (*a*–*d*). Scale bar in (*e*) represents 20 µm in (*e–i*). (*j*) Pyd suppresses Dx-induced signalling in S2 cells. S2 cells were transfected with empty vector (EV) or as indicated. Normalized mean Notch reporter activity measured by luciferase reporter expression is relative to cells expressing Notch only. Error bars are s.e.m., *n* = 3, * or ** indicates *p* < 0.05 by Student *t*-test for comparisons as indicated. (*k*) Pyd does not suppress ligand-induced signalling. Luciferase assay in S2 cells showing the consequence of Pyd expression on ligand-induced Notch activation. Normalized mean Notch reporter activity is shown relative to cells expressing Notch co-cultured with non-expressing S2 cells. S2 cells were transfected as indicated. Data represent mean of five experimental repeats, each performed in triplicate. Error bars are s.e.m., * indicates *p* < 0.05, Student *t*-test for comparison as indicated on graph.

In the wing imaginal disc, Notch signals at the boundary of the dorsal and ventral wing compartments to induce *wingless* expression and regulate wing margin formation [21]. Notch signalling can be induced independently of its ligands by the overexpression of Dx in the wing imaginal disc [2]. The latter results in ectopic *wingless* expression in the imaginal discs which is reflected in extra wing margin sensory bristles in the adult wing (figure 1*a,b,e,f*). We found that *pyd* mutants result in a strong increase in these Notch activation phenotypes, exhibiting increased levels of ectopic *wingless* expression and consequently a higher number of ectopic sensory bristles (figure 1*c,g*). Dx activates an endosomal Notch activation pathway that depends on the late endosomal HOPS component *car* [2]*.* When Dx is expressed in a *car* mutant background the ectopic signalling is suppressed and there is additionally a downregulation of endogenous Notch activity with reduced *wingless* expression (figure 1*d,h*). Thus Dx can act negatively on Notch if the latter's trafficking to the late endosome is suppressed. We therefore investigated whether the upregulated Dx-induced signal resulting from *pyd* mutation was also sensitive to loss of *car*. Expressing Dx in a *car, pyd* double mutant background also blocked ectopic Notch signalling, and further exhibited clear gaps in the expression of *wingless* at the dorsal–ventral boundary of the imaginal discs (figure 1*i*). Thus loss of Pyd upregulates the late endosome-dependent Notch signal.

To further investigate the action of Pyd on Dx-induced Notch signalling, we used a previously established cell culture assay that enables us to monitor signalling by using a Notch responsive reporter construct driving luciferase expression [4]. In our cell culture assays we are able to measure Dx-induced signalling separately from any ligand-induced component depending on whether Notch is coexpressed with Dx or if Notch expressing cells are presented with ligand-bearing cells [4]. As previously shown [4], the coexpression of Dx with Notch upregulated Notch signalling observed in S2 cells. The additional expression of Pyd reduced this Dx-induced signal (figure 1*j*). In contrast Pyd expression did not have any significant effect on Notch activity initiated by exposure to ligand-bearing cells (figure 1*k*).

### 2.2. Pyd and Dx regulate cap cell recruitment to the germline stem cell niche

**Figure 2.**
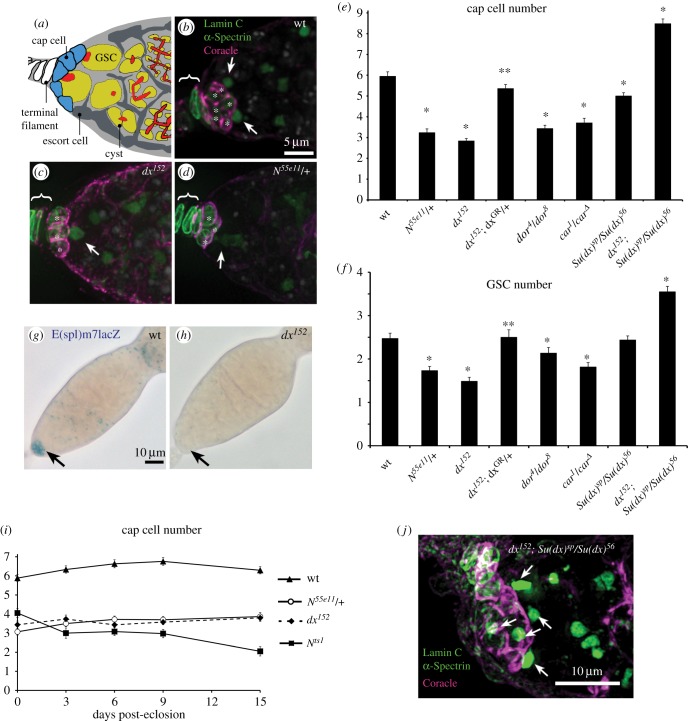
Dx regulates the size of the ovary niche. (*a*) Diagram of the *Drosophila* GSC niche showing terminal filament (white), cap cells (blue) and escort cells (dark grey). GSCs (yellow) are attached to cap cells and contain the spectrosome at their anterior membrane (red). Dividing cysts (yellow) show the branching fusome (red) extending through their cytoplasm. (*b–d*) Germaria labelled for DAPI (grey), anti-Coracle (magenta) strongly marking cap cell membrane and weakly terminal filament and escort cells, anti-α-Spectrin marking the GSC spectrosome (arrows, green) and Lamin C weakly marking cap cells and but more strongly the TF (also green). Images (*b–d*) represent a merged stack of deconvolved layers. (*b*) wt niche. (*c*) *dx^152^* and (*d*) *N^55e11^*/+ niches showing reduced number of cap cells (asterisks) and GSCs compared to wt. (*e,f*) *dx^152^* and *N^55e11^* have reduced number of cap cells (*e*) and GSCs (*f*) compared to wt. *dx^152^* phenotype is rescued by genomic rescue construct (dx^GR^). HOPS complex mutants, *dor* and *car* mutations also reduce niche size and stem cell numbers. * and ** indicate *p* < 0.05 by two tailed Mann–Whitney *U* test, respectively compared with wt and *dx^152^*. (*g,h*) *E(spl)m7*-lacZ expression seen in wt cap cells (arrow) (*g*) is reduced in *dx^152^* (*h*). (*i*) Adult cap cell number for *dx^152^* and *N^55e11^*/+ over time since eclosion is stable but cap cell numbers reduce when strong Notch loss of function is induced by shift of temperature-sensitive N^ts1^ to 29°C as previously reported [22]. Error bars (*e,f,i*), s.e.m. (*j*) expanded niche of *dx^152^*; *Su(dx)^sp^/Su(dx)^56^* ovaries, arrows indicate associated spectrosome-containing GSCs. Scoring of cap cell and GSC numbers for this genotype is shown in (*e,f*).

To examine the physiological relevance of the Pyd/Dx functional interaction we examined genetic interactions between these components *in vivo*. Notch signalling has been shown to control niche size during development, as it is activated in the somatic cell niche precursors by Delta ligand expressed in the terminal filament. The precursor cells differentiate as cap cells that constitute the niche, which is subsequently populated by 2–3 germline stem cells (GSCs) per ovariole (figure 2*a*) [22–24]. Pyd has previously been identified as a negative regulator of Notch signalling in the cap cells of the *Drosophila* ovary GSC niche. *Pyd* mutants display an increased Notch signalling level and increased niche size [7]. In contrast we found that *dx* mutants have the opposite phenotype showing reduced niche size, populated by fewer GSCs (figure 2*a-c,e,f*), an outcome similar to the removal of one copy of the *Notch* gene (figure 2*d,e,f*). Addition of a Dx genomic rescue construct (Dx^GR^, TX05B) [25] reverted the *dx* phenotypes (figure 2*e,f*). We next investigated a Notch reporter, *E(spl)M7-lacZ* [7], and found reduced Notch signalling in the cap cell niche of *dx* mutants (figure 2*g,h*). A key distinguishing feature of the Dx-dependent component of Notch activation is the requirement for HOPS complex components such as Carnation (Car)/VPS33 and Deep orange (Dor)/VPS18 which are involved in late endosomal/lysosomal fusion [2,4,26]. We found that mutations in these components also reduced niche size (figure 2*e,f*).

In the adult niche Notch also plays a role in niche maintenance [22–24]. When Notch activity is switched off in the adult using a temperature-sensitive mutant (*N^ts1^*), the size of the niche declines due to loss of cap cells (figure 2*i*) [22–24]. To determine the contribution of *dx* to these distinct Notch functions, adults were dissected immediately after eclosion and at various adult ages up to 15 days. In *dx* null flies, the niche size was reduced in newly eclosed flies but remained stable as adult flies aged (figure 2*i*). Thus loss of *dx* specifically reduces cap cell recruitment but does not affect subsequent adult niche maintenance. Flies heterozygous for a null allele of *Notch* also eclosed with a similarly reduced niche size, which also did not further decline with age (figure 2*i*). Thus the developmental role of Notch in niche formation is more sensitive to reduction of signalling activity, making the contribution of Dx more critical during this phase. Because of previously reported close functional relationship between Dx and Su(dx) activities, we investigated how the *dx* phenotype was affected by mutations removing Su(dx). *Su(dx)^sp^/Su(dx)^56^* mutant ovaries showed only a weakly reduced niche size compared to wild-type controls but maintained a similar GSC number (figure 2*e,f*). However, we found that in flies double mutant for *dx* and *Su(dx)* the phenotype of both mutants was reversed, instead producing an increased niche size (figure 2*e,f,j*). We have previously reported similar reversals in the direction by which Dx acts on Notch contingent on Su(dx) gene copy number, which likely reflects opposing activities on the ligand-dependent and -independent modes of Notch activation [4].

**Figure 3.**
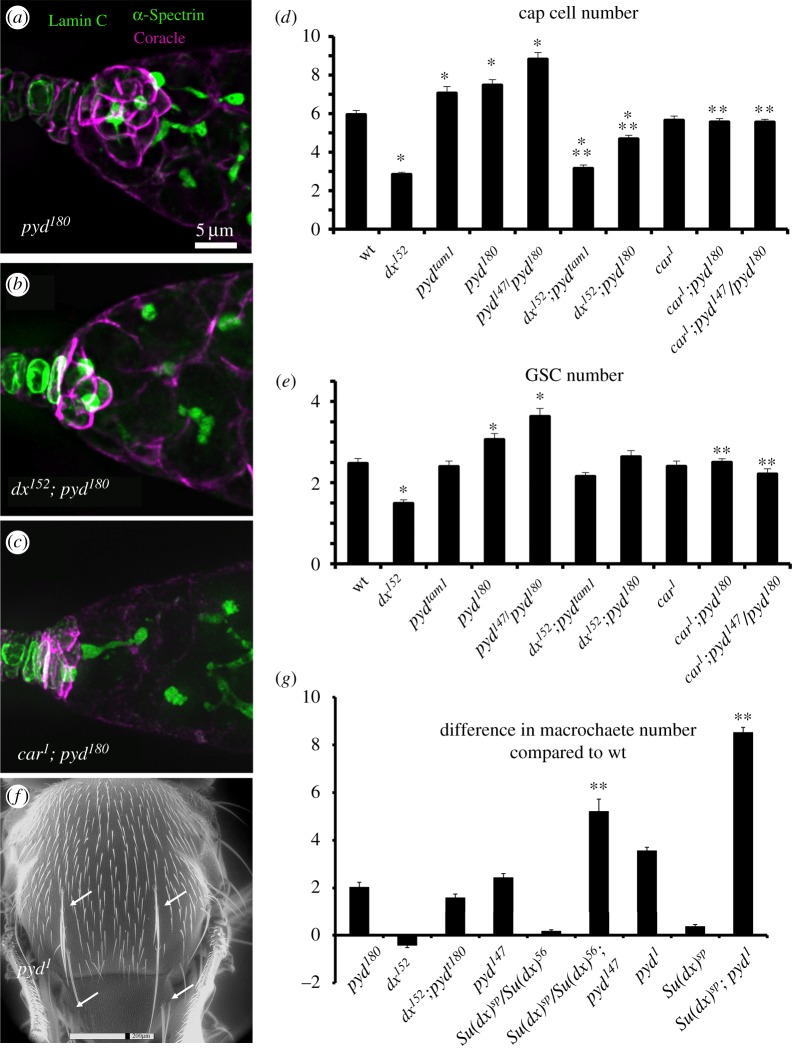
Pyd antagonizes Dx function in the ovary stem cell niche. (*a–c*) shows the GSC niche region of ovarioles stained for anti-Coracle (magenta), anti-Lamin C (green) and anti-α-Spectrin (also in green, marking the GSC spectrosomes). (*a*) *pyd^180^* has an enlarged GSC niche; this is reduced to below wild-type levels in flies additionally mutant for *dx^152^* (*b*). The hypomorphic *car^1^* also reduces the *pyd* expanded niche phenotype (*c*). The scale bar in (*a*) represents 5 µm in (*a–c*). (*d*) Genetic interactions between *pyd* and *dx* or *car* mutations. *pyd* mutants have more cap cells than wild-type. *dx^152^*, *pyd* double mutants have cap cell numbers below the wild-type number. *car^1^* also prevents the *pyd* niche expansion phenotype. (*e*) GSC number is reduced by the *dx* mutation and increased by the stronger *pyd* mutant alleles. GSC number of *pyd* mutants is significantly reduced by *car^1^*. * and ** indicate *p* < 0.05 by two tailed Mann–Whitney *U* test respectively compared to wt and comparative *pyd* mutations combinations; *n* = 14 to 48 individuals scored for each genotype. (*f*) environmental scanning EM of *pyd^1^* mutant fly indicating ectopic macrochaetae phenotype on notum and scutellum (arrows). (*g*) Scoring of macrochaetae phenotypes as difference compared to wild-type. ** indicates *p* < 0.05 for comparison with appropriate *pyd* mutant genotype (Student *t*-test, *n* = minimum of 25 flies scored for each genotype. Error bars (*d,e,g*), s.e.m.

Having established the requirement for Dx-dependent Notch signalling in the full development of the niche, we next investigated genetic interactions with *pyd* mutants. The *pyd* mutants displayed an increased number of cap cells and higher GSC numbers for the stronger alleles. We found that introducing a mutation of *dx* reduced the increased niche size of *pyd* mutant ovarioles and reduced the number of GSCs although the latter was not statistically significant (figure 3*a,b,d,e*). However the *car^1^* mutation significantly rescued both the expanded *pyd* niche and GSC phenotypes (figure 3*c,d,e*). Thus Pyd normally opposes a Notch signal in the GSC niche that depends on late endosome trafficking, consistent with a model in which ZO-1 acts negatively on the Dx-dependent signalling pathway. In contrast, *dx* mutants did not have a significant effect on the *pyd* bristle phenotype (figure 3*f,g*). Furthermore, while a suppressive interaction of *Su(dx)* with *pyd* mutants occurs in the ovary niche [7], the *Su(dx)* mutation enhanced the bristle gain phenotype of null *pyd* mutants (figure 3*f,g*). The differences in the interplay between these mutations suggests an alternative regulatory network involving these components is likely involved in developmental contexts where Pyd acts positively to support Notch signalling levels instead of negatively as it does in the ovary stem cell niche.

### 2.3. Pyd alters Dx-regulated Notch trafficking in cells

**Figure 4.**
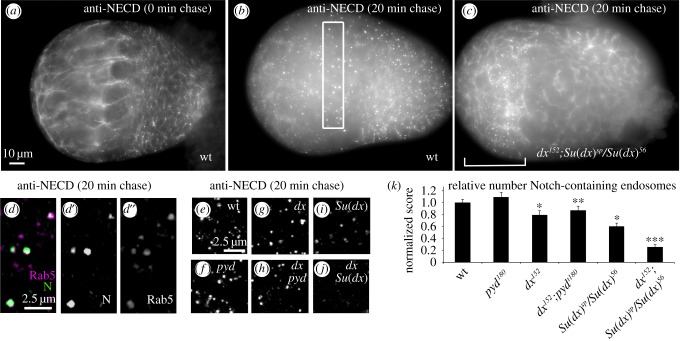
Notch endocytic uptake assay in developing ovary. (*a*) 0 h after puparium formation, wt ovary showing Notch staining with anti-NECD with 0 min chase displaying cell surface Notch localization. (*b*) Wild-type ovary, after 20 min chase. Notch localization is predominantly in endosomal locations. Box represents region just posterior to terminal filaments used for scoring of Notch-containing endosomes in (*e–k*). (*c*) A *dx^152^*; *Su(dx)^sp^/Su(dx)^56^* ovary after 20 min chase showing mostly cell surface localization and few Notch containing endosomes, apart from terminal filament region indicated by bracket. (*d*) Merged image showing localization of Notch (magenta) in Rab5 positive (green) endosomes after 20 min chase. (*d',d''*) show single channel images of Notch and Rab5 respectively. (*e–j*) Representative 50 layer merged images to illustrate the differences in endocytosis between the indicated mutant backgrounds. (*k*) Scoring of mean number of Notch-containing endosomes per standardized unit volume, counted using ImageJ and normalized to wt. * indicates *p* < 0.05 compared to wt, ** indicates *p* < 0.05 compared to *pyd^180^*, *** indicates *p* < 0.01 compared to *dx^152^* or compared to *Su(dx)^sp^/Su(dx)^56^*, two tailed Mann–Whitney, *n* = 8 to 22. Error bars, s.e.m.

We next investigated how Pyd might regulate Dx-activated Notch signalling. Dx has been shown to act by promoting entry of Notch into an endocytic trafficking pathway [2–4] and we wondered if *pyd* mutants might increase the amount of Notch endocytosis. This suggestion is somewhat contrary to a previous report of an accumulation of adherens junction Notch in *pyd* mutants in wing imaginal discs, which might imply that Notch endocytosis is reduced in a *pyd* mutant [7]. However this apparent accumulation of Notch may more reflect apical domain expansion rather than decreased trafficking [7]. Indeed we found that endocytic uptake of Notch in *pyd* mutant ovary tissue was not impaired (figure 4), although we were not able to detect a statistically significant increase in the number of Notch containing endosomes compared to wild-type. However, since it was not straightforward to quantitate the total amount of Notch present in the endosomes and because Notch can enter the cell by multiple mechanisms, both Dx-dependent and independent [4], we reasoned that our *in vivo* assays may not be sufficiently specific to detect *pyd* mutant outcomes affecting a subset of endocytosed Notch, although at least a proportion of Notch endocytosis occurring in both wild-type and *pyd* mutant backgrounds is Dx-dependent (figure 4*k*). This redundancy of Notch entry routes is highlighted by the consequences of removing Dx and Su(dx) function. While *dx* and *Su(dx)* mutants each alone resulted in moderate reduction of Notch endocytosis, when the mutants were combined there was an additive effect resulting in loss of most Notch containing endosomes and a corresponding clear surface localization of Notch even after a 20 min endocytic chase period (compare figure 4*b* and *c*). Increased ligand-induced signalling through the surface accumulated Notch is the likely explanation for the enlarged niche phenotypes observed in the double mutant case. Notably the loss of endocytosis observed in the *Su(dx)* and *dx* double mutant tissue was regionally dependent, as within the terminal filament region (figure 4*c*) a substantial amount of Notch-containing endosomes was observed, indicating that still further means by which Notch can be internalized into the cell remain to be characterized.

**Figure 5.**
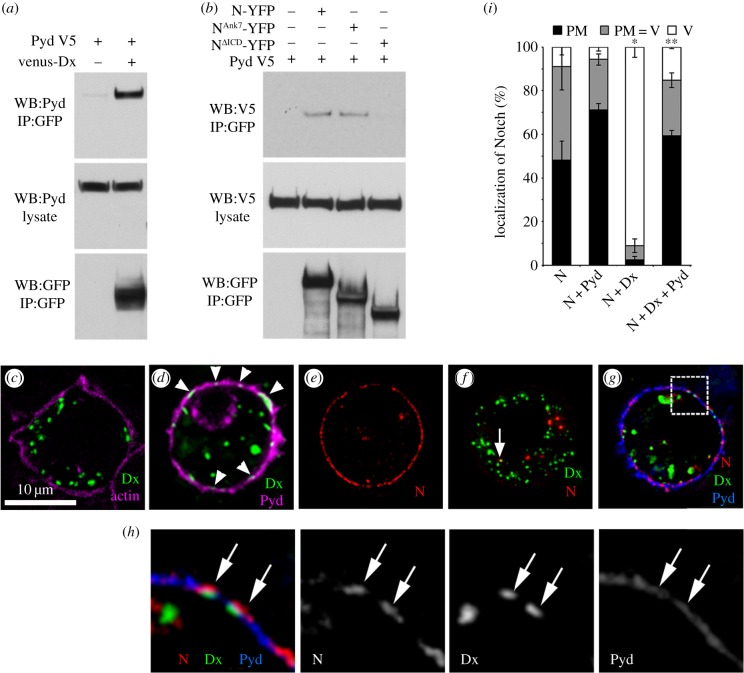
Pyd interacts with Dx to antagonize Dx-induced Notch trafficking. (*a*) Coimmunoprecipitation of Dx and Pyd. S2 cells were transfected as indicated. Venus-tagged Dx was immunoprecipitated using GFP-TRAP beads and detected using anti-GFP. Pyd protein was detected using anti-Pyd. (*b*) Notch and Pyd coimmunoprecipitate. YFP-tagged Notch proteins were immunoprecipitated using GFP-TRAP beads and detected using anti-GFP. V5-tagged Pyd was detected using anti-V5. Interaction requires Notch intracellular domain but the region C-terminal to Notch Ankyrin repeats is dispensible for complex formation. (*c,d*) Recruitment of Dx to plasma membrane by Pyd in S2 cells. (*c*) S2 cells were transfected with venus-Dx (green) and costained for actin (magenta), (*d*) venus-Dx (green) cotransfected with Pyd-V5 (magenta). Arrowheads in (*d*) indicate Dx puncta at the plasma membrane that are seen only with Pyd coexpression. Scale bar, 10 µm. (*e-i*) Consequence of Pyd expression on Notch endocytic trafficking assayed by uptake of bound anti-NECD antibody. (*e*) Notch expressed alone in S2 cells is mostly localized to cell surface after 1 h chase. (*f*) Co-expression of Dx induces endocytic localization of Notch, with Notch only colocalizing with Dx in a small proportion of endosomes after a 1 h chase (arrow). *(g)* When additionally coexpressed with Pyd, Notch is retained at the plasma membrane. Box in (*g*) is magnified in (*h*) to show colocalization of Notch, Dx and Pyd at plasma membrane (arrows in *h*), separate channels shown in greyscale. (*i*) Notch ECD antibody localization after 1 h chase in each cell was scored as % cells displaying plasma membrane (PM), vesicular (V), or intermediate (PM = V) Notch localization. Data show mean % localization from three separate experiments. Each experimental repeat quantified Notch localization in a minimum of 80 cells. * indicates *p* < 0.001 compared to cells transfected with Notch only, ** indicates *p* < 0.001 compared to cells transfected with N and Dx. Statistical significance determined by two tailed Mann–Whitney *U* test. Error bars are s.e.m.

**Figure 6.**
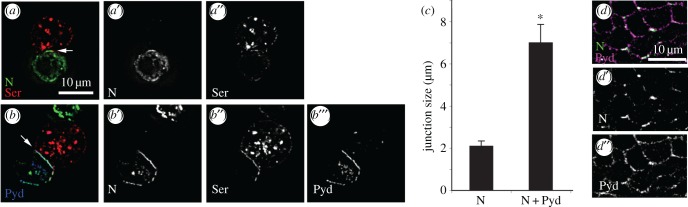
Pyd increases cell–cell contact size of Notch and Serrate (Ser) expressing cells. (*a*) Notch-Ser co-clustering is observed when Notch-expressing cells (green) and Ser-expressing cells (red) were mixed (arrows). (*b*) Co-clustering was promoted when Notch cells were expressing GFP-tagged Pyd resulting in longer junctional interfaces. (*a,b*) Merged pictures of Notch (green), Ser (red) and Pyd (blue). Notch staining (*a',b'*), Ser staining (*a″,b″*) and Pyd-V5 staining (*b*′′′) are shown separately (grey scale). (*c*) Quantified mean junction length of Notch/Ser interface. *n* = 27–35, error bars, s.e.m., * indicates *p* < 0.05, Student *t*-test). (*d*) Immunostaining showing colocalization of Notch (green) and Pyd (magenta) at discrete locations around cell boundaries in 0 hours APF ovary. (*d',d''*) show single channels in grey scale.

To investigate the consequences of Pyd on Notch in a simpler experimental system, we used a cell-culture model in which the source and route of Notch endocytosis can be controlled by the expression of specific regulatory components [4]. To determine whether Pyd and Dx exist in a complex we used a coimmunoprecipitation assay and found that Pyd coprecipitates with Dx (figure 5*a*). We similarly found an interaction between Pyd and Notch (figure 5*b*). When coexpressed in S2 cells, some Dx staining puncta became colocalized in discrete foci at the cell surface together with Pyd, a surface localization not observed when Pyd was not expressed (figure 5*c,d*). To investigate the mechanism by which Pyd might block Dx-induced Notch signalling, we performed a pulse chase Notch endocytosis assay [4] using antibodies to the Notch extracellular domain. When Notch is expressed alone in S2 cells it is largely localized at the cell surface (figure 5*e*). Expression of Dx greatly increases Notch internalization into the endocytic pathway (figure 5*f*). Although Pyd has a wide distribution around the cell periphery, we found that Notch, Pyd and Dx are coincident at discrete foci at the cell membrane (figure 5*g,h*) and Pyd expression reduces Notch endocytosis (figure 5*i*). Pyd also colocalized with Notch in the absence of Dx and this was particularly evident when Notch was clustered at cell contacts when forming junctions with ligand-bearing cells in co-culture experiments (figure 6*a,b*). Indeed Pyd expression increased the junction length of ligand/Notch contact regions (figure 6*c*), and this may explain the small, although not statistically significant, increase in ligand-induced signalling observed in cell signalling assays (figure 1*k*). Notch and Pyd also colocalized at discrete, punctate locations at cell–cell contacts *in vivo* (figure 6*d*).

**Figure 7.**
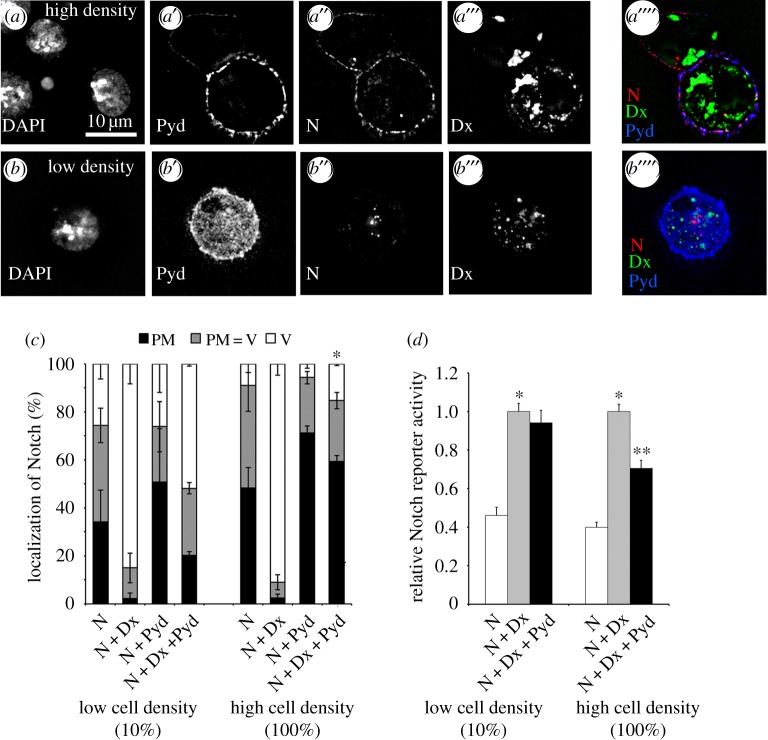
Cell density modulates Pyd regulation of Notch. (*a–c*) Notch uptake assay at low and high cell density. Transfected cells were confluent (*a*, high cell density) or diluted 1/10 with medium (*b*, low cell density) before reseeding, and left 24 h before performing Notch antibody uptake endocytosis assay. (*a,b*) Representative images of Notch uptake assay at respectively high and low cell density. DAPI, Pyd, Notch and Dx staining are shown in greyscale in (*a–a'' and b–b''*) respectively. (*a"',b"'*) Merged staining of Notch (red), Dx (green) and Pyd (blue). Pyd is recruited to cell membrane at high cell density. (*c*) Notch ECD antibody localization after 1 h chase in each cell was scored as plasma membrane (PM), vesicles (V), or intermediate (PM = V). Pyd blocks Dx-induced Notch endocytosis at high cell density but has little effect at low cell density. Data show mean % localization from three separate experiments, error bars are s.e.m.. For each experimental repeat Notch localization was classified in a minimum of 80 cells. Pyd blocks Dx-induced Notch endocytosis at high cell density but is less effective at low cell density. * indicates *p* < 0.001 for mean % vesicular localization compared to N + Dx + Pyd at low cell density. (*d*) S2 cells were transfected as indicated and seeded at low or high density. Normalized mean Notch reporter activity is shown relative to cells expressing Notch and Dx. * indicates *p* < 0.001 compared to N only. ** indicates *p* < 0.002 compared to N + Dx. Experiments were performed a minimum three times, each with technical triplicates. Error bars, s.e.m. Statistical significance determined by two tailed Mann–Whitney *U* test.

A characteristic feature of mammalian ZO-1 activity is that it has previously been shown to mediate cell density-dependent signal regulation [18,19,27]. We wondered whether Dx-induced Notch signalling would be similarly modulated. Pyd was only strongly localized to the cell membrane at high cell densities, and we found that Pyd expression was less effective at reducing Dx-induced Notch endocytosis at low cell density and also did not significantly suppress Dx-induced Notch signalling (figure 7*a–d*). Dx mediated signalling, at least in a cell culture context, can therefore be regulated upstream by the cell's external environment through Pyd surface accumulation. Thus in principle Pyd regulation of Notch may act as an alternative means by which external cues can tune the Notch signal output by Dx-regulated Notch trafficking.

## 3. Discussion

Notch signalling can be activated by ligand-dependent or -independent mechanisms [2–4,28]. The latter can be induced by the activity of Dx and, unlike ligand-induced signalling, this mode has a strict requirement for late endosomal Notch trafficking. Although it appears that the Dx-promoted activity of Notch can contribute to full Notch signalling levels during development, it has not been clear whether this alternative form of signalling can itself be modulated in response to upstream inputs. Scaffold adaptor proteins play key roles in enabling cross talk between junctional complexes and signalling pathway components through multiple binding sites [29]. ZO-1 is a conserved adaptor protein that associates with adherens and tight junctions [30]. Pyd, the *Drosophila* ZO-1 homologue, acts both positively and negatively on Notch signalling depending on developmental context [7]. Here we uncover a mechanism by which Pyd can downregulate Notch activity by suppressing the ligand-independent Dx-driven mechanism of Notch signal activation. Thus Pyd can both regulate Notch signalling levels and select between alternative means of its activation.

We found that Pyd could form a complex with both Dx and Notch and can block signalling by reducing Dx-induced Notch endocytosis. At present the molecular mechanism by which this block on endocytosis occurs is unclear. It is possible that binding prevents an interaction of some unidentified accessory protein, or Notch is sequestered in a membrane protein complex with ZO-1 in a way that prevents Notch assembly into clathrin-coated vesicles. This may represent a similar activity to that previously reported for mammalian ZO-1, whose dissociation from the gap junction complex is linked to the latter's endocytic internalization into the cell [31,32]. In cell culture we found that the ability of full-length Pyd to block Dx-induced endocytosis and signalling also depended on high cell density whereupon Pyd became localized to the cell surface. Thus these cells can sense their local environment to regulate Notch independently of its ligands through Pyd.

To determine if there was a functionally relevant interaction between Pyd and Dx *in vivo* we investigated genetic interactions between the genes in ovary stem cell niche development. Pyd-dependent suppression of Notch signalling is normally required during the assembly of the GSC niche in the *Drosophila* ovary to limit niche size [7]. The ovary GSC niche assembles following the larval-pupal transition, after the formation of the terminal filament stacks that prefigure ovariole formation. At this time somatic cells intercalate between the terminal filaments and the germline progenitor cells, and are recruited to differentiate as cap cells which exit mitosis and subsequently provide anchorage for the GSCs. Notch signalling is required in the cap cells for their recruitment by the terminal filament, and up or downregulation of Notch increases or reduces niche size respectively [22–24]. Delta is expressed in the terminal filament and ligand-stimulated Notch signalling contributes to cap cell recruitment and niche formation. Despite this we found Dx is also required for full niche assembly during development. Furthermore, *pyd* mutants increase Notch signalling and niche size and this is dependent on Dx and also Carnation, a HOPS complex component required for Dx but not ligand-induced Notch activation [2]. Thus we infer that the ligand-independent signalling mode also contributes to the establishment of sufficient cells with cap cell identity to generate the full niche size and normal GSC population level. We further speculate that assembly of cell–cell contacts and the associated junction proteins during assembly of the germline niche ultimately helps determine the population size of the GSCs by dampening this ligand-independent signalling component. We should note however that our *in vivo* data do not rule out the possibility that there may be additional functions for Pyd in the niche that are not Dx-dependent. The population of the niche by GSCs may depend on a number of factors including the number of cap cells and adhesiveness of GSC/cap cell contacts, as well as the morphology of the niche, which may explain why effects of mutant combinations on GSC number were not as clear in some mutant combinations compared to cap cell number.

Recent work has shown that another apical localized protein, Crumbs, can also act to suppress a Dx-dependent Notch activity in the wing disc through an extracellular domain, which also suppresses endocytosis [33]. This recently published work differs from ours in that they observed a visible increase in Notch endocytosis in *crumbs* mutant clones in wing disc tissue. The endocytic removal of Notch in *crumbs* mutants is not prevented by loss of Dx but instead Notch accumulates in the apical region of the cell. They infer that Dx in this case is required for further progression of Notch through the endocytic pathway. This is curious because *dx* mutants in an otherwise wild-type background cause clear surface accumulation of Notch at the membrane of imaginal wing disc epithelial cells and Dx overexpression causes increased Notch endocytosis. We and others have shown previously that Dx can act in two locations in the cell, to promote Notch endocytosis and to direct Notch to the late endosomal limiting membrane [2–4]. It is possible therefore that Pyd and Crumbs act on separate initial endocytic entry routes into the cell, respectively Dx-dependent and -independent, but downstream the endocytic flux converges on Dx-dependent activation.

In the light of the above discussion it is interesting to contemplate the relationship between Su(dx), Dx and Pyd in the different contexts in which Pyd acts negatively and positively on Notch. In the niche, Su(dx) loss of function appears to increase Pyd activity such that one copy of Pyd is sufficient to produce a normal size niche [7]. The effect of *Su(dx)* mutation could thus indirectly be antagonistic to Dx-promoted Notch activity since Pyd itself opposes Dx function. However Su(dx) also acts directly on Notch and competes with Dx activity to divert Notch into an alternative endocytic internalization route [4]. Thus in this case *Su(dx)* mutations could be expected to increase Dx efficacy. This balance of opposing activities may explain why *Su(dx)* mutants alone have little effect on niche size. Interestingly, when flies are simultaneously homozygous for both *dx* and *Su(dx)* mutants there is a phenotypic switch to an increased niche size. Thus both Dx and Su(dx) can each function to either promote or antagonize niche size depending on the genetic background in which they are placed. We have observed similar reversals of phenotype before and attribute this behaviour to the fact that both genes act negatively on ligand-induced Notch signalling [4]. Hence removing both endocytic entry routes increases the availability of Notch for ligand-induced activation, which outweighs any reduction in ligand-independent signalling that results from loss of *dx*. In this light it is interesting to contemplate that the expanded niche phenotypes arising from the different mutant causes reported here are associated with different Notch sub-cellular distributions and this is likely linked to different cellular locations of Notch activation.

In the peripheral nervous system, where Pyd has the opposite activity to promote Notch activity [7], we found that *dx* mutants did not significantly suppress the *Pyd* phenotype and the latter was enhanced by loss of *Su(dx)* rather than suppressed. Thus in this case Su(dx) and Pyd may act cooperatively through an unknown mechanism to suppress bristle formation. It is possible that in this Notch-promoting context Pyd function acts to stimulate ligand-dependent Notch signalling. Although we did not observe direct evidence for the latter in cell culture Notch reporter luciferase assays, we did find that Pyd promoted Notch/ligand contacts at cell interfaces. It is possible that this *in vitro* observation may reflect an activity that normally promotes ligand-dependent signalling in an *in vivo* context where cells are apically polarized and form adherens junctions.

Our results therefore illustrate the complexity by which Notch regulation is integrated into the morphology and functioning of the cell, with endocytic networks interfacing with protein networks controlling cell architecture and cell interactions to tune signalling to physiologically appropriate levels. The complexity of these networks is hence revealed in the often counterintuitive phenotypic outcomes of different combinations of mutants that we observe.

## 4. Material and methods

### 4.1. *Drosophila* stocks

All experiments were at 25°C on standard *Drosophila* culture medium. *car^1^*, *N^55E11^* (Bloomington Stock Center), *dx^152^* [34], *dx^GR^ TX05B* [25], *Su(dx)^sp^*, *Su(dx)^56^* [35], *E(spl)m7-lacZ*, *pyd^147^, pyd^180^* [7], *pyd^tamou^ (tam1)* [5]; *carΔ* [26], *y^1^w^67c23^* (Bloomington Stock Center) was used as wild-type (wt)**.**

### 4.2. S2 cell culture

S2 cells were grown at 25°C in Schneider's medium (Invitrogen), with 10% FBS (Hyclone) and antibiotics. S2 cells were transfected using standard Effectene (Qiagen) or CaCl_2_ methods. Constructs: pUAST-Pyd-GFP as described previously [7]. Pyd constructs were also subcloned into pMT (Invitrogen), removing GFP and inserting a C-terminal V5-tag. pMT-Notch-YFP, pMT-Notch, pMT-NotchΔIntra-YFP, pMT-Notch-Ank7-YFP were derived from pUASTNotch-YFP (gift of K. Matsuno). pMT-NotchΔIntra-YFP and pMT-Notch-Ank7-YFP were generated by removing the intracellular domain, or the region after Ankyrin repeat 7 respectively. pMT-Venus-Dx and pMT-Dx-V5 were derived from pUAST-Venus-Deltex (gift from K. Matsuno). Other vectors were pUAST-EYFP-Rab7 (gift from M.P. Scott), pUAST-Ser and H/N (Heat-Shock inducible Notch) [36,37], and pMT-GAL4 (*Drosophila* Genomics Resource Center, Indiana).

### 4.3. Immunocytochemistry and histology

Primary antibodies were mouse anti-Notch extracellular domain (C458.2H, concentrate, 1 : 200, DSHB); mouse anti-Notch intracellular domain, (C17.9C6, concentrate, 1 : 1000, DSHB); rabbit anti-V5 (1 : 1000, Bethyl Laboratories); goat anti-Serrate (1 : 1000, Santa Cruz); guinea pig anti-Coracle (D4.1.3, 1 : 5000, R. Fehon); mouse anti-Lamin C (LC28.26, 1 : 20, DHSB), mouse anti-αSpectrin (3A9, 1 : 10, DSHB), rabbit anti-Pyd (1 : 2000) [7], rabbit anti-Rab5 (ab31261, 1 : 500 Abcam). Actin staining was performed with Alexa Fluor^®^ 647 Phalloidin (1 : 100, Thermo Fisher Scientific).

Ovarioles were immunostained as previously described [23], except primary antibody incubation was at room temperature. S2 cells were grown on poly-lysine (Sigma) coated coverslips. Immunostaining was performed at room temperature. Cells were fixed in 4% formaldehyde (Polysciences) for 30 min, rinsed in PBS, permeabilized in PBS/Triton X-100 (PBS-tx) 0.2%, and blocked for 1 h in 3% skimmed milk/PBS, then incubated with primary antibody in blocking solution for 2 h, and washed in PBS before 1 h incubation with secondary antibody. Tissue and cell preps were washed in PBS-tx 0.1% and mounted in Vectashield with DAPI (Vector Labs). For cell culture Notch endocytic uptake assay, S2 cells grown on coated coverslips were incubated with C458.2H for 15 min on ice, washed with ice cold S2 medium and chased for 60 min at 25°C. Cells were fixed, permeabilized, and stained as above. Aggregation assay with Notch (expressed from H/N vector) and ligand (pUAST-Ser/pMT-Gal4) expressing cells was as described [36]. Images were captured using Volocity (Perkin Elmer) with an Orca-ER digital camera (Hamamatsu) mounted on a M2 fluorescent microscope (Zeiss). Deconvolution of 0.5 µm optical sections was performed with three nearest neighbours using Openlab (Improvision), or by iterative deconvolution (Volocity) and processed in Photoshop (Adobe). Distance measurements were performed with ImageJ.

For endocytic uptake assays in ovary tissue, preps were dissected at 0 h after puparium formation (APF) in ice cold Graces (Sigma) and incubated with anti-NECD (C458.2H, concentrate, DSHB) at 1 : 200 in 200 µl PBS in PCR tubes on ice. The sealed PCR tubes were placed in a 7 ml tube containing ice and gently rotated for 1 h at 10 r.p.m. in a cold room. The contents of the PCR tube were then transferred to a small sieve and rinsed three times with ice cold Graces followed by three 5 min washes in ice cold Graces. The preps were transferred to prewarmed Graces at 25°C for 20 min chase and then fixed for 20 min with 4% formaldehyde, washed three times with PBS-tx 0.1%, and permeabilized for 2 h in PBS-tx 0.3% and 4% normal donkey serum (NDS) (Jackson). The preps were incubated for 2 h in PBS + 4% NDS + anti-Vasa rat (1 : 300, S. Levine), washed in PBS-tx 0.1% three times for 5 min then three times for 15 min and treated with corresponding fluorescent-conjugated secondary antibodies. Preps were mounted between spacers, to avoid crushing, in Vectashield antifade Mounting Medium with DAPI (Vector Labs). Each prep was imaged with the same exposure using Z sections separated by 0.5 µm, and deconvolved with Openlab using the same parameters. Endosomal Notch-containing structures were counted using ImageJ in the region adjacent to the terminal filaments containing the nascent cap cell progenitors. A standardized area of 64 × 11.6 µm was scored for each section and the scores of 10 adjacent Z sections were used to derive a mean score for each 10 layer series. Three to five such sections were processed for each ovariole and a mean score for each ovariole calculated. All data were normalized to y^1^w^67c23^.

### 4.4. Immunoprecipitation and western blotting

For coimmunoprecipitation experiments, S2 cells were grown in 6-well dishes and transfected with pMT plasmids. CuSO_4_ was added after 24 h to induce expression and after a further 24 h cells were homogenized in lysis buffer (50 mM Tris–HCl, pH 8.0, 150 mM NaCl, 1% Triton X-100, 1 mM CaCl_2_) and protease inhibitor cocktail (EDTA-free Complete; Roche), and cleared by centrifugation. The lysate was incubated with 10 µl GFP-TRAP agarose (Chromotek) for 1 h at 4°C, and washed four times in lysis buffer. Bound proteins were eluted with sample buffer, run on 3–8% Nupage Gels (Invitrogen) and western blotted with rabbit anti-Pyd (1 : 10 000) [7], mouse anti-V5 (1 : 5000, Invitrogen) or rabbit anti-GFP (1 : 20 000, ImmunoKontact).

### 4.5. Notch luciferase reporter assay

S2 cells in 12-well dishes were transfected when approximately 50% confluent with combinations of pMT-Notch, pMT-DxV5, pMT-Pyd, NRE:Firefly (gift from S. Bray) and Actin:Renilla (gift from G.Merdes). After 24 h, when cells reached 100% confluence, they were re-suspended and seeded into white 96-well plates (Nunc #136101) and CuSO_4_ was added after a further 24 h. 24 h after induction, luciferase activity was assayed with Dual-Glo Luciferase (Promega), quantified by a luminometer (Berthold), and Firefly/Renilla ratio was calculated for triplicate samples. For cell-density experiments, cells were re-seeded into white 96-well plates as above (high cell density) or diluted 1/10 with culture medium before reseeding (low cell density). For ligand-induced signalling assays, Notch expressing cells were layered on top of fixed Delta expressing S2 cells (S2-Mt-Dl; DGRC) according to the previously described protocol [4]. Experiments were repeated a minimum of three times.
